# Clusterin and Its Role in Insulin Resistance and the Cardiometabolic Syndrome

**DOI:** 10.3389/fimmu.2021.612496

**Published:** 2021-02-25

**Authors:** Jennifer Wittwer, David Bradley

**Affiliations:** Division of Endocrinology, Diabetes and Metabolism, Department of Internal Medicine, Diabetes and Metabolism Research Center, The Ohio State University, Columbus, OH, United States

**Keywords:** adipocyte, clusterin, cardiometabolic disease, type 2 ddiabetes mellitus, inflammation

## Abstract

The cardiometabolic syndrome involves a clustering of metabolic and cardiovascular factors which increase the risk of patients developing both Type 2 Diabetes Mellitus and cardio/cerebrovascular disease. Although the mechanistic underpinnings of this link remain uncertain, key factors include insulin resistance, excess visceral adiposity, atherogenic dyslipidemia, and endothelial dysfunction. Of these, a state of resistance to insulin action in overweight/obese patients appears to be central to the pathophysiologic process. Given the increasing prevalence of obesity-related Type 2 Diabetes, coupled with the fact that cardiovascular disease is the number one cause of mortality in this patient population, a more thorough understanding of the cardiometabolic syndrome and potential options to mitigate its risk is imperative. Inherent in the pathogenesis of insulin resistance is an underlying state of chronic inflammation, at least partly in response to excess adiposity. Within obese adipose tissue, an immunomodulatory shift occurs, involving a preponderance of pro-inflammatory immune cells and cytokines/adipokines, along with antigen presentation by adipocytes. Therefore, various adipokines differentially expressed by obese adipocytes may have a significant effect on cardiometabolism. Clusterin is a molecular chaperone that is widely produced by many tissues throughout the body, but is also preferentially overexpressed by obese compared lean adipocytes and relates strongly to multiple components of the cardiometabolic syndrome. Herein, we summarize the known and potential roles of circulating and adipocyte-specific clusterin in cardiometabolism and discuss potential further investigations to determine if clusterin is a viable target to attenuate both metabolic and cardiovascular disease.

## Introduction

Although the exact diagnostic criteria varies (1–3), the metabolic syndrome involves a clustering of abnormalities including obesity, insulin resistance, hypertension, and dyslipidemia. These in turn heighten the risk of cardio- and cerebrovascular disease (CVD) [elevated risk of primary and recurrent stroke (4) and myocardial infarction (5)], Type 2 Diabetes Mellitus (T2D) (6, 7), and non-alcoholic fatty liver disease/steatohepatitis (NAFLD/NASH) (8). Initially termed the metabolic syndrome, Reaven's syndrome, or Syndrome X, among others (9, 10), the ramifications of metabolic disease on CVD risk have subsequently led to a broadening of terminology (i.e., the cardiometabolic syndrome). Although the criteria are the same (Table 1), the term cardiometabolic syndrome has gained more widespread acceptance due to the intersection of risk factors that contribute to both CVD and metabolic disease and involve similar pathophysiologic processes.

**Table 1:** Clinical definitions of the cardiometabolic syndrome based on the National Cholesterol Education Program (NCEP) Expert Panel on Detection, Evaluation, and Treatment of High Blood Cholesterol in Adults Adult Treatment Panel III, the International Diabetes Federation (IDF), and the World Health Organization (WHO).

	**WHO (11)**	**NCEP ATP III (2)**	**IDF (12)**
	T2D or IFG or IGT or insulin resistance plus ≥ 2 of the following:	3 of the following:	Central obesity defined as WC above the ethnicity-specific cut-off plus ≥ 2 of the following:
Body Weight	• BMI > 30 kg/m2 or WHR > 0.85 (females) or > 0.90 (males)	• WC > 88 cm (females) or > 102 cm (males)	Population specific
Lipid Profile	• HDL <1.0 mmol/L (<40 mg/dL) and/or • TG ≥ 1.7 mmol/L (150 mg/dL)	• HDL <1.3 mmol/L (<50 mg/dL) and/or • TG ≥ 1.7 mmol/L (150 mg/dL)	• HDL <1.3 mmol/L (<50 mg/dL) or specific treatment and/or • TG ≥ 1.7 mmol/L (150 mg/dL) or specific treatment
Blood pressure	• BP ≥ 140/90 mmHg or use of blood pressure medication	• BP ≥ 135/85 mmHg or use of blood pressure medication	• BP ≥ 135/85 mmHg or use of blood pressure medication
Other	• Microalbuminuria > 20 pg/min or Alb/Crea ratio ≥ 30 mg/g		• Fasting plasma glucose ≥ 5.6 mmol/L (100 mg/dL) or previously diagnosed T2D

*BP, blood pressure; HDL, high density lipoprotein cholesterol; IGT, impaired glucose tolerance; T2D, type 2 diabetes; TG, triglycerides; WC, waist circumference; WHR, waist to hip ratio*.

The cardiometabolic syndrome is highly prevalent, affecting over 30% of the adult population in the United States (U.S.) and rising, with especially high prevalence rates (>40%) in patients older than 60 years old (13, 14). Compared to the general population, the relative risk for developing CVD with coexistent cardiometabolic syndrome is doubled (15), with 3-fold the risk of T2D (13). In addition, all-cause mortality is higher in those with the cardiometabolic syndrome. Importantly, factors related to ethnicity/race, gender, and socio-economics affect risk, with the highest rates occurring in non-Hispanic white men and black women (14). In addition, socio-economic factors such as low education level and advanced age are independently associated with a higher risk of the cardiometabolic syndrome. The reasons for these differences are incompletely understood and likely multifactorial, but remain a critical focus of future research with significant public health ramifications (16–19).

## The Central Role of Obesity-Related Inflammation and Insulin Resistance in Cardiometabolism

Over 35% of the adult US population is obese (20), and excess adiposity contributes to multiple complications including T2D and accelerated rates of CVD (21). In fact, CVD is the number one cause of mortality in diabetic patients, with a 2–3-fold higher risk of clinical atherosclerosis (22), illustrating a close association between metabolic disease and CV risk. As such, underlying the dysfunction in cardiometabolic disease are four interrelated central features: insulin resistance, excess visceral adiposity, atherogenic dyslipidemia, and endothelial dysfunction (23). Of these, obesity-related insulin resistance appears to the most important trigger. Among all the cardiometabolic risk factors, the relationship between insulin resistance and hypertension is the best established, and end-organ insulin resistance is a central tenet in its pathophysiology (24). Various mechanisms have been put forth to explain this connection including a decrease in insulin-mediated renal artery vasodilatation and uncompensated sodium reabsorption, with a resultant increase in blood pressure. Systemic and vascular insulin resistance occurs in conjunction with inappropriate activation of the renin–angiotensin–aldosterone system (RAAS) (25). Hyperinsulinemia also increases sympathetic nervous system activity (26), contributing further to the development of hypertension, a prominent component of the cardiometabolic syndrome.

Obesity and its associated comorbidities (including T2D and CVD) are associated with a state of chronic low-grade inflammation (27) that is well-recognized as a major cause of decreased insulin sensitivity (28–30). Inflammatory pathway activation has been observed in all classical insulin target tissues, indicating the key role of inflammation in driving the pathogenesis of systemic insulin resistance. Particularly, in adipose tissue (AT), macrophages play a central role (28, 31, 32); however, recent studies have highlighted the importance of several other key immune cells in maintaining lean AT, including immunosuppressive regulatory T (T_reg_) cells, which contribute to a “Type 2” anti-inflammatory immunoenvironment (33, 34). In obesity, this immunologic milieu is shifted to a more pro-inflammatory state, in which the normal architecture, energy storage, and endocrine activities of adipocytes are profoundly altered. Activation of a proinflammatory pathway in AT leads to the secretion of numerous cytokines such as tumor necrosis factor-alpha (TNF-α), interleukin-6 (Il-6) and interleukin-1β (IL-1β) (35) that activate toll-like receptors (TLR2 and TLR4) and impair glucose uptake (36). Cytokines also impair suppression of AT lipolysis, with resultant free fatty acid (FFA) release into the circulation (37–39), which hinders the ability of insulin to stimulate muscle glucose uptake (40) and suppress hepatic glucose production (41), the two major factors in the pathogenesis of insulin resistance. Therefore, disruption in AT fatty acid metabolism is likely an underlying factor in cardiometabolic disease, by promoting both hyperglycemia and dyslipidemia.

Obesity, the cardiometabolic syndrome, and T2D have also long been associated with higher risk of cerebrovascular disease and cognitive decline (42–52). One potential reason for this connection is that insulin has direct effects on neurotransmission and neuropathology in the brain (53–56), including alterations in the production, degradation and clearance of β-amyloid (Aβ) that lead to plaque deposition in Alzheimer's disease (57). Various murine models of obesity and diabetes (including after high-fat diet feeding) (58–61) have indicated a relationship between peripheral and “central” insulin resistance, and in humans altered metabolic brain activity occurs in peripherally insulin-resistant subjects (62–64), with dysregulation in CNS insulin signaling (65–67). In fact, intravenous insulin infusion (57, 68, 69), inhaled insulin (69, 70), the insulin-sensitizing agent pioglitazone (70, 71), metformin (72, 73), and weight-loss interventions, including bariatric surgery, have demonstrated beneficial effects on memory (74–77). Cerebrovascular disease (78–80) and vascular dementia (81, 82) are also strongly related to insulin resistance, even independent of frank diabetes, and the Insulin Resistance Intervention after Stroke (IRIS) trial established that improving insulin sensitivity can prevent cerebrovascular events (83).

## Characteristics of Clusterin and Physiologic Roles

The human clusterin (*CLU*) gene (encodes the protein clusterin/apolipoprotein J) was first identified by Blaschuk et al. (84). This highly conserved gene consists of nine exons located on chromosome 8 that encode different isoforms resulting from alternative splicing and post-translational modifications (glycosylation, disulfide bond cleavage, etc.) (85, 86). The *CLU* gene promoter is highly conserved among species, with numerous identified regulatory elements including TGF-β inhibitory element, activator protein-1 and−2, and nuclear factor, but is also responsive to many environmental and cytokines that vary depending on the involved tissue (87–89). Although expressed by nearly every tissue in the human body, clusterin is predominantly made by epithelial tissues during embryonic development and in the testis, ovary, adrenal gland, liver, heart and brain of adults (85, 86). Its identified receptors are varied and often tissue-specific and include the HDL cholesterol receptor, low density lipoprotein-related protein 2 (LRP/megalin) (90), ApoER2 (91), and very low density lipoprotein receptor (VLDLR), many of which are critical to cardiovascular health.

There are two major forms of clusterin: a stress-induced, non-glycosylated, nucleocytostolic 55kDa variant (nCLU) consisting of parallel α and β chains, and a secreted or cytosolic variant (sCLU) that is proteolytically cleaved, connected by five disulfide bonds, and released from cells in an antiparallel fashion (92). Heterodimeric sCLU circulates mainly as a component of high-density lipoprotein (HDL) cholesterol, but has also been found to be bound to apolipoprotein (Apo) A1, various lipids, paroxanase, beta (β)-amyloid protein, and complement proteins, among others [summarized in Trougakos and Gonos (93)]. In healthy subjects, a higher prevalence of sCLU is bound to cardioprotective HDL cholesterol, suggesting that secreted clusterin may play a role in preventing progression of vascular disease (94). In contrast, nCLU predominantly promotes ionizing radiation-induced death of cells and triggers apoptosis in a BAX-dependent mechanism, and has yet to be linked with cardiometabolic pathology (95). Therefore, the remainder of this review will focus on the relationship of CVD and metabolic disease with sCLU.

One of the major roles of clusterin is to act as a molecular chaperone that assists folding of secreted proteins (87). Clusterin may also serve as a sensor of oxidative stress and is reduced upon exposure to acute stress (96). As a result of its ubiquitous nature, it has been implicated in a wide range of pathologic processes including cancer development and progression, complement regulation, and sperm maturation (93, 97, 98). *CLU* gene transcription and protein expression is upregulated in breast cancer (99), ovarian cancer (100), and prostate cancer (101), and inhibition of *CLU* expression protects the cell from apoptosis induced by chemotherapy, radiotherapy, and androgen/estrogen depletion (102–104). Clusterin is also involved in CNS lipid trafficking (105, 106) and is widely expressed in the brain (107). Accordingly, clusterin has clinical associations with Alzheimer's disease (AD) (108, 109) and has been proposed as a biomarker of AD (110). In fact, risk variants in *CLU* are strongly associated with AD (108). In patients with both mild cognitive impairment and AD, clusterin levels are elevated in the brain, cerebrospinal fluid, and blood (111–114), and accordingly *CLU* gene expression is elevated in these pathologic conditions (107).

## Role of Circulating Clusterin in Insulin Resistance and Metabolic Disease

There are numerous identified mechanisms by which circulating clusterin could impact the risk of metabolic disease. Leptin resistance has been demonstrated in both murine models and human obesity, with reduced transport across the blood-brain-barrier (BB) (115). In turn, sCLU affects the transport of leptin across the BBB via LDL cholesterol (116), and through its binding to the receptor LRP2 can sensitize leptin receptors in the hypothalamus (117). This suggests that clusterin may play a role in modulating appetite and contributing to obesity (117). Clusterin can also directly affect insulin signaling and inflammation, two factors that can lead to insulin resistance, via its actions on macrophage phosphoinositide 3-kinase (PI3K; a mediator of insulin signaling) and NFκB (a major pro-inflammatory pathway in insulin resistance) (118). Clusterin induces directional migration of macrophages acting as a chemoattractant (119). This stimulates the expression and secretion of TNF-α and various chemotactic cytokines allowing clusterin to serve as a link between inflammation and remodeling of tissues by directing immune cells (120). Therefore, clusterin plays a significant role in inflammation and immune responses through its molecular interactions with complement factors, immunoglobulins, and inflammatory pathways (121).

In support of these identified mechanistic processes, both murine and human studies have demonstrated a significant link between circulating clusterin and features of the metabolic syndrome. Skeletal muscle and hepatic gene expression of *CLU* increase following high-fat diet feeding in mice, and whole body clusterin knockout mice are insulin sensitive compared to wild-type mice (122). Obese patients without diabetes following a 2 week very low calorie diet have reduced plasma clusterin levels (123), and in obese compared to lean subjects, plasma clusterin levels are elevated and positively relate to body mass index, waist circumference, markers of inflammation (hsCRP and retinol-binding protein-4) (124), and insulin resistance (125). In addition, polymorphisms in *CLU* have been linked to insulin resistance [by the homeostasis model of insulin resistance [HOMA-IR] and impaired insulin secretion [HOMA-β]] (126). In contrast to these deleterious metabolic effects, clusterin has been shown to reduce hepatic fibrosis via stellate cell downregulation of the Smad3 signaling pathway (127).

## Cardiovascular and Cerebrovascular Effects of Circulating Clusterin

The mechanistic effects of circulating clusterin on CVD are controversial, due to seemingly paradoxical effects in the existing literature, and the mechanisms behind such a link remain unclear. Clusterin is found in a subset of dense HDL cholesterol particles and has wide-ranging effects on lipid transport (121, 128). In plasma, clusterin forms HDL particles with ApoA-I and ApoE and aids in the transfer of HDL cholesterol from peripheral tissues to the liver, diverting lipoproteins away from atherosclerotic lesions (129, 130). In contrast, clusterin may have a deleterious effect on the antioxidant activity of paroxanase-1 (PON1), whose deficiency enhances atherosclerosis by increasing the accumulation of oxidized phospholipids in atherosclerotic plaques (131).

There are multiple lines of evidence suggesting that human clusterin may have a significant clinical association with multiple facets of cardiovascular risk. Circulating plasma clusterin (sCLU) levels are strongly associated with the pro-inflammatory factor C-reactive protein (CRP) (124), various lipid markers of heightened cardiovascular risk, and increasing systolic and diastolic blood pressure (90, 132). Circulating clusterin is also negatively associated with leptin in obesity-related CVD (133). In addition, clusterin bound to HDL cholesterol is reduced in obese males and is associated with lower levels of HDL cholesterol, higher TGs (134) and low-density lipoprotein (LDL) cholesterol levels, and accelerated atherogenesis (135), and may confer higher cardiovascular risk during the aging process (135). Interestingly, proteomic analysis has shown that higher levels of clusterin are found in carotid atherosclerotic compared to non-atherosclerotic plaques (136). Not all studies, however, have confirmed a beneficial role for clusterin in CVD. A recent study showed that lower serum clusterin was associated with higher rates of mortality in heart failure patients (137), indicating some uncertainty on the importance of circulating clusterin in the CVD process.

## Adipocyte-Derived Clusterin and Its Potential Role in Cardiometabolic Disease

The adipocyte is no longer viewed as simply a storage depot for lipids, but is now recognized as an important determinant of an obesity-related proinflammatory environment, instigating inflammation in expanding AT (138). Despite significant progress in our understanding of the role of the adipocyte as an immumodulator, and evidence that circulating plasma and HDL cholesterol bound clusterin may be involved in the metabolic syndrome, insulin resistance, atherogenesis, and CV risk, the importance of adipocyte-derived clusterin in human cardiometabolic disease remains largely unknown. In whole human AT, *CLU* gene expression is higher in obese compared to lean subjects, and is decreased following weight loss induced by VLCD or bariatric surgery (123). We have recently shown that clusterin derived specifically from the adipocyte may play an important role in cardiometabolic disease (90). In obese compared to lean human subjects, adipocyte gene expression and protein levels of clusterin were higher and responsive to (FFA) palmitate stimulation (a major component of a high fat diet enriched in fatty acids) (139). In addition, we found strong associations of adipocyte clusterin with systemic insulin resistance, multiple components of the metabolic syndrome (HDL cholesterol, the ratio of HDL cholesterol to total cholesterol, and TGs, and both systolic and diastolic blood pressure), and overall CVD risk and mortality. In this same study, clusterin treatment of human liver cells reduced insulin signaling by lowering Akt phosphorylation and promoting key genes involved in gluconeogenesis; yet hepatic expression of the major regulator of hepatic *de novo* lipogenesis [sterol regulatory element-binding protein-1 [*SREBP-1*]] and *APOA1* were decreased in response to clusterin binding to LRP2. These results suggest that the liver receptor LRP2 may be a key target for the potential cardiometabolic role of clusterin. Knockdown of *SREBP-1* can perpetuate hyperglycemia via enhanced gluconeogenesis and reduced glycolysis and glycogen synthesis (140). APOA1 is a major protein associated with HDL cholesterol particles in plasma which facilitates efflux of cholesterol from cells, notably from macrophages within atherosclerotic plaques, to the liver for excretion. Low plasma APOA1 levels are also a strong predictor of CVD (141). In a mouse model prone to non-alcoholic steatohepatitis (NASH) adipocyte *CLU* expression also paralleled an increase in liver fat, hepatic fibrosis, and steatohepatitis (90).

**Figure 1:**
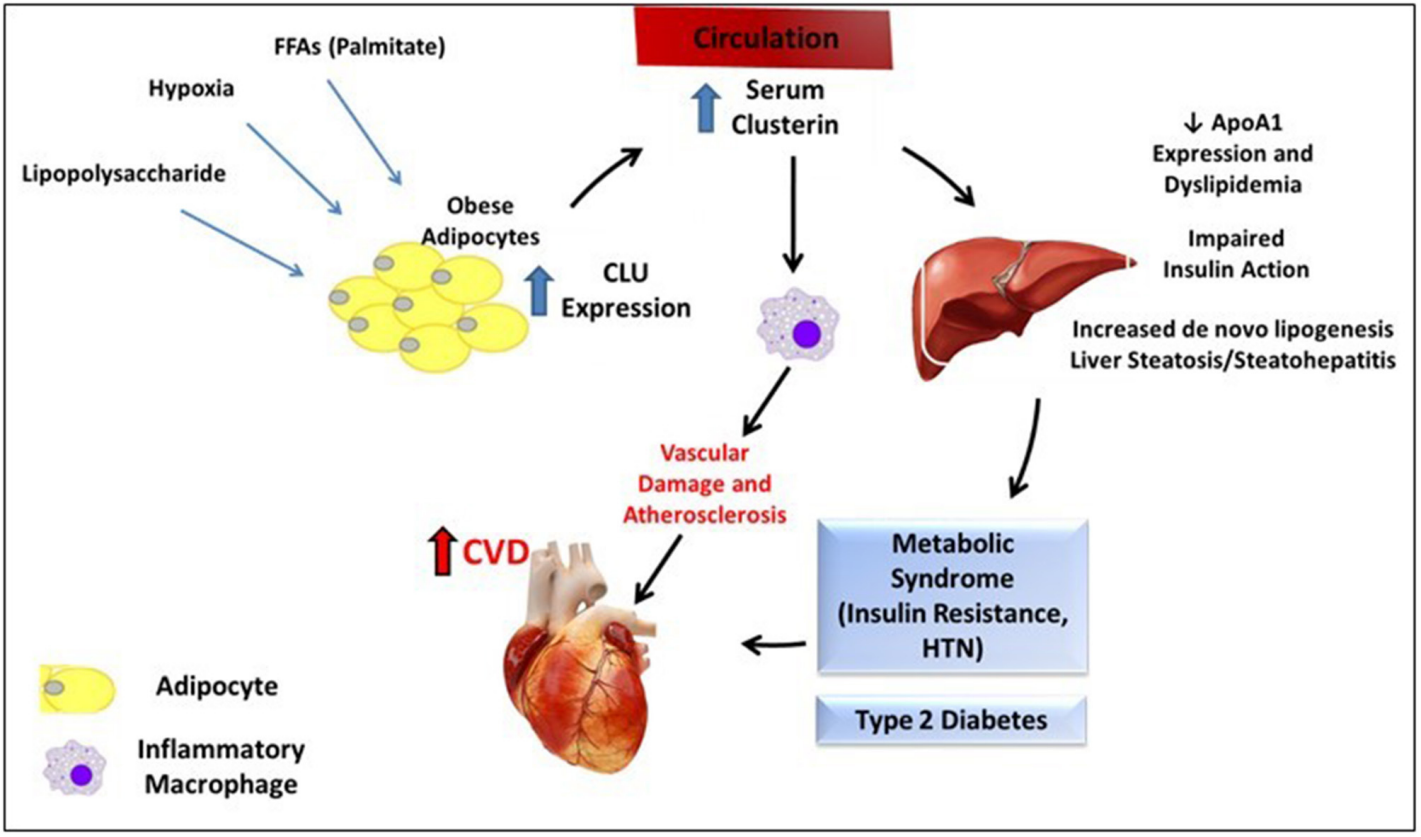
Summary of proposed mechanism for clusterin-mediated cardiometabolic disease. Various stimuli may increase adipocyte expression of CLU from adipocytes in the setting of obesity. Circulating clusterin subsequently has multiple effects on the liver (reduction in ApoA1 expression, dyslipidemia, impaired insulin signaling, and potentially increased steatosis and inflammation) and on macrophages, which may contribute to the cardiometabolic syndrome, and increase CVD risk.

Although these results suggest several mechanisms by which clusterin could link insulin resistance, metabolic disease, and CVD (Figure 1), further investigation is needed to fully elucidate the cardiometabolic role of AT clusterin, and specifically clusterin derived from the adipocyte. Although treatment with the FFA palmitate stimulates clusterin release *in vitro*, other potential triggers for clusterin expression are possible. These include AT hypoxia, which has previously been shown to increase clusterin expression in other cell types outside of AT (142). In addition, the effects of adipocyte-derived clusterin on the AT immunoenvironment and the skewed balance of pro- and anti-inflammatory cytokines observed in human obesity is also unknown.

## Conclusion

The cardiometabolic syndrome is a clustering of metabolic and cardiovascular abnormalities that increase the risk of CVD, T2D, and all-cause mortality. The rising prevalence of the cardiometabolic syndrome, both in the U.S. and worldwide, make a more thorough understanding of its pathophysiologic underpinnings imperative. Although likely multifactorial, the presence of obesity-related insulin resistance appears to be a central, if not instigating factor. Systemic and tissue-specific insulin resistance not only affect endothelial function and leads to atherogenic dyslipidemia, but propagate a pro-inflammatory environment that includes excess release of detrimental FFAs into the circulation. Clusterin is a ubiquitous protein secreted by many organs/tissues throughout the body. Although studies have implicated circulating clusterin in multiple metabolic and cardio/cerebrovascular abnormalities, a unifying mechanism remains elusive, and the current literature is inconsistent and inconclusive. In particular, the importance of AT derived clusterin, strongly associated with many metabolic and CVD risk factors, requires further investigation. This includes understanding the exact mechanistic processes by which it acts locally within AT and systemically in the liver, endothelial cells, and the vasculature. Isolating its effects, potentially through the development of adipocyte-specific clusterin knockout and overexpression models, will be instrumental in determining if it is a viable target to attenuate features of the cardiometabolic syndrome.

## Author Contributions

JW and DB co-wrote the manuscript. Both authors contributed to the article and approved the submitted version.

## Conflict of Interest

The authors declare that the research was conducted in the absence of any commercial or financial relationships that could be construed as a potential conflict of interest.
